# Sugar Consumption Produces Effects Similar to Early Life Stress Exposure on Hippocampal Markers of Neurogenesis and Stress Response

**DOI:** 10.3389/fnmol.2015.00086

**Published:** 2016-01-19

**Authors:** Jayanthi Maniam, Christopher P. Antoniadis, Neil A. Youngson, Jitendra K. Sinha, Margaret J. Morris

**Affiliations:** ^1^Department of Pharmacology, School of Medical Sciences, University of New South Wales AustraliaSydney, NSW, Australia; ^2^Endocrinology and Metabolism Division, National Institute of Nutrition, Indian Council of Medical ResearchHyderabad, India

**Keywords:** early life stress, limited nesting, sucrose, glucocorticoid receptor, neurogenic differentiation 1, DNA methylation

## Abstract

Adverse early life experience is a known risk factor for psychiatric disorders. It is also known that stress influences food preference. We were interested in exploring whether the choice of diet following early life stress exerts long-lasting molecular changes in the brain, particularly the hippocampus, a region critically involved in stress regulation and behavioral outcomes. Here, we examined the impact of early life stress induced by limited nesting material (LN) and chronic sucrose availability post-weaning on an array of hippocampal genes related to plasticity, neurogenesis, stress and inflammatory responses and mitochondrial biogenesis. To examine mechanisms underlying the impact of LN and sugar intake on hippocampal gene expression, we investigated the role of DNA methylation. As females are more likely to experience adverse life events, we studied female Sprague-Dawley rats. After mating LN was imposed from days 2 to 9 postpartum. From 3 to 15 weeks of age, female Control and LN siblings had unlimited to access to either chow and water, or chow, water and 25% sucrose solution. LN markedly reduced glucocorticoid receptor (*GR*) and neurogenic differentiation 1 (*Neurod1*) mRNA, markers involved in stress and hippocampal plasticity respectively, by more than 40%, with a similar effect of sugar intake in control rats. However, no further impact was observed in LN rats consuming sugar. Hippocampal *Akt3* mRNA expression was similarly affected by LN and sucrose consumption. Interestingly, DNA methylation across 4 CpG sites of the *GR* and *Neurod1* promoters was similar in LN and control rats. In summary, early life stress and post-weaning sugar intake produced long-term effects on hippocampal *GR* and *Neurod1* expression. Moreover we found no evidence of altered promoter DNA methylation. We demonstrate for the first time that chronic sucrose consumption alone produces similar detrimental effects on the expression of hippocampal genes as LN exposure.

## Introduction

Adverse early life experience, commonly known as early life stress, has increasingly been demonstrated to constitute an important risk factor for the emergence of psychiatric-related phenotypes (Heim et al., 1997, 2010; Heim and Nemeroff, 2002). This is thought to be partly due to early life stress modifying the expression levels of genes governing behavior and response to stress (Avishai-Eliner et al., 2002). Diet is another factor which can impact behavioral responses such as anxiety, stress response and memory. Foods that are rich in either sugar or fat have been shown to be able to relieve stress and anxiety in both human and animal studies (Prasad and Prasad, 1996; Willner et al., 1998; Desmet and Schifferstein, 2008). However, importantly, animal work reveals that a high energy diet leads to hippocampal dependent memory deficits (Molteni et al., 2002; Kanoski et al., 2007; Beilharz et al., 2014) but its impact on hippocampal gene expression is less known. The majority of these observations were made in male rats, and there are limited data on diet-induced memory deficits in females. Moreover, its interaction with early life stress is yet to be explored. Given the complexity of the brain and compensatory adaptation following early life stress exposure (Maniam and Morris, 2012; Maniam et al., 2014), examining the consequences of poor diet in addition to early life stress experience on hippocampal gene expression will provide insights into mechanisms and targets for intervention. This is particularly relevant given the increased risk for mental health disorders in those previously exposed to early-life stress (Heim and Nemeroff, 2001, 2002).

The hippocampus is key in regulation of hippocampal-dependent memory and is highly susceptible to early-life stress (Bremner et al., 1997). There is substantial evidence demonstrating that early life stress alters hippocampal structure and function in addition to hippocampal gene expression (Brunson et al., 2003; Fenoglio et al., 2006). Consistent findings in rodents show that early life stress induced by disruption of the mother-child relationship alters several molecular pathways that regulate the stress response (Ladd et al., 2000; Maniam and Morris, 2010a,b), neurogenesis and plasticity (Hulshof et al., 2011; Lajud et al., 2012) and serotonin (Own et al., 2013). Recent evidence shows an association between early life stress and brain mitochondrial dysfunction. For example, rats that were prenatally stressed exhibited reduced expression of hippocampal *Pgc-1*α, master regulator of mitochondrial biogenesis (Glombik et al., 2015; Stachowicz et al., 2015). Despite the mitochondria playing an integral role in cell growth and differentiation, there appears to be very limited work on the effect of postnatal stress on genes involved in mitochondrial biogenesis. Therefore, here the expression of relevant hippocampal genes involved in mitochondrial biogenesis was also measured.

There has been recent growing interest in the impact of diets that are in high fat and sugar, or sugar alone, on molecular changes in the hippocampus, with respect to understanding diet-related hippocampal dependent behavioral deficits, particularly related to cognition. While there is evidence from human studies showing an impact of high fat and sugar diet intake on memory tasks (Øverby et al., 2013; Nyaradi et al., 2014), examining the direct effect of the diet on hippocampal gene expression in human is not plausible. Work in animals has shown that diet can have a direct effect on the hippocampus. In rats, a diet high in sugar, or fat and sugar combined, when consumed acutely (5 days) or chronically was shown to impair hippocampal dependent, but not non-hippocampal tasks, and induced marked metabolic changes (Kanoski and Davidson, 2010; Beilharz et al., 2014). These findings suggest that the hippocampus is particularly sensitive to dietary insult. These dietary effects on memory were measured in males, and whether similar effects occur in females is unknown. Moreover, the consumption of such foods is detrimental to the structural and functional integrity of the hippocampus (Kanoski and Davidson, 2011) and also for synaptic plasticity and neurogenesis (Lindqvist et al., 2006; Stranahan et al., 2008). In agreement, diets enriched with sugar alone or combined high fat and sugar consumed acutely or chronically promotes the expression of inflammation-related genes (Boitard et al., 2014; Sobesky et al., 2014), and reduces the expression of genes involved in neurogenesis and neuroplasticity (Molteni et al., 2002).

Only a few animal studies have examined the impact of consuming a post-weaning diet high in fat and sugar following early life stress. These studies mainly focused on the behavioral consequences and selected hippocampal gene expression. For example, the consumption of a high fat and high sugar diet reversed the increased anxiety-like and depression-like behavior related to early life stress induced by maternal separation, which was associated with normalization of hippocampal *GR* mRNA, and this was observed in both male and females (Maniam and Morris, 2010a,b). In female rats, early life stress induced by limited nesting has been shown to increase the preference for foods rich in fat and sugar but corresponding molecular changes in the brain were not examined (Machado et al., 2013). In the context of stress interaction with diet, a study that examined the combined effects of psychosocial stress with high fat diet intake found an exaggerated deficit in hippocampal dependent tasks vs. stress or diet alone (Alzoubi et al., 2009). Hence, the combined effect of early life stress and a poor diet may be deleterious to the hippocampus.

The interaction between post-weaning diet and early life stress-induced hippocampal gene changes warrants attention for several reasons. Firstly, understanding the implications of these combined factors is essential as early life stress and poor diet often co-exist in the modern world. Importantly, the extent to which later environment and lifestyle contribute to psychopathology risk during development in those exposed to a stressful early environment remains unknown. As we are living in a constantly changing environment, it is important to examine how lifestyle impacts the consequences of early life stress. Further, chronic stress influences the preference and intake of foods which are cheap and easily accessible (Maniam and Morris, 2012; Morris et al., 2014). For example, the consumption of energy rich, palatable food is often used as a coping strategy by those under stressful conditions (Adam and Epel, 2007). The increased preference for high sugar and fat foods during stressful experiences in healthy individuals is well documented (Adam and Epel, 2007). In animal studies, acute stress exposure in normal rats increases intake or preference for high fat and sugar diet (Dallman et al., 2005; la Fleur et al., 2005).

The robust hippocampal gene expression changes induced by early life stress have been shown to be associated with DNA methylation in a great number of studies (McGowan et al., 2009; Murgatroyd et al., 2009; Roth et al., 2009; Naumova et al., 2012). There is growing evidence that expression of key hippocampal genes, particularly GR and BDNF, can be regulated by altered DNA methylation, as documented both in human (McGowan et al., 2009; Naumova et al., 2012) and animal (Roth et al., 2009) work.

These questions are difficult to address in humans, where it is impossible to control for long term confounders such as lifestyle and nutrition, or to examine underlying mechanisms, and thus robust and relevant animal models are essential. Several models of early life stress have been tested so far, however, the LN paradigm resembles human maternal neglect, where the mother is present but care is fragmented (Ivy et al., 2008). Hence this model was utilized in the current study to simulate early life stress. Here we examined the combined impact of early life stress and chronic sucrose availability post-weaning on an array of hippocampal genes related to plasticity, stress regulation, mitochondrial biogenesis, inflammatory response and serotonin. Further, we assessed whether a sucrose diet implemented at weaning would moderate the gene transcription effects of early life stress. To understand the mechanisms underlying the impact of LN and sugar intake on hippocampal genes, we further investigated the role of DNA methylation in regulating hippocampal gene expression in this LN paradigm. As women appear to have an increased risk of suffering from stressful life events and seem to be more susceptible to the development of mental illnesses such as major depression (Chapman et al., 2004; Dinan, 2005), our study focussed on female rats.

## Methods

### Subjects

All animal procedures were approved by the Animal Care and Ethics Committee of UNSW Australia. Male and female Sprague-Dawley rats (Animal Resource Centre, Perth, WA, Australia) were maintained in a temperature controlled (23°C) colony room on a 12 h light/dark cycle (lights on at 0700 h) with *ad libitum* access to standard laboratory chow and water. Mating was carried out in house; litters comprising 9–15 pups were included and standardized to 12 pups per litter on postnatal day (PND) 1 using pups from dams littering on the same day to minimize alterations in maternal behavior and pup nutrition. Litters were housed with the dam in polypropylene cages (20 × 32 × 19 cm) on wood shavings with a metal lid. On PND 2 litters were assigned to either normal bedding (control) or LN. As previously described (Maniam et al., 2015a,b), the LN paradigm involved fitting a metal base to the cage, which was raised slightly to allow urine and droppings to escape. Rats in the LN group were provided with a single piece of paper towel as bedding material. Both groups were left undisturbed between PND 2–9, following which LN dams received normal bedding material until weaning.

### Post-weaning diet

At PND 21, pups were weaned and housed 3–4 rats per cage. Female offspring were assigned to either: standard laboratory chow (11 kJ/g energy; 12% fat, 21% protein, 65% carbohydrate, Gordon's Specialty Stockfeeds, NSW, Australia) and access to two water bottles; or chow and access to one bottle containing sucrose (25% w/vol) and one water bottle. Chow, water and sucrose were available *ad libitum*. This generated 4 groups: Control+Chow (Con-Chow); Control+Sucrose (Con-Sucrose); LN+Chow (LN-Chow) and LN+Sucrose (LN-Sucrose). Rats were weighed at weaning and at regular intervals throughout the study. Food and fluid intake over 24 h was recorded at regular intervals by weighing food and bottles before presentation to the rats and again after 24 h. Energy intake per cage was measured and average intake was calculated based on supplier's information.

### Terminal tissue collection

At 15 weeks of age, rats were anesthetized by ketamine/xylazine (Ketamine: 100 mg/ml Xylazine: 20 mg/ml, dose: 100/15 mg/kg i.p.), killed by decapitation and the brain was removed immediately. The whole hippocampus was dissected and snap frozen in liquid nitrogen then stored at −80°C. Both right and left hippocampal lobes were ground for determination of mRNA expression and DNA methylation of genes of interest.

### Reverse-transcription quantitative PCR (qRT-PCR)

Hippocampal RNA was extracted using Tri-reagent (Sigma-Aldrich, Sydney, NSW, Australia) and treated with DNase I (Invitrogen, Melbourne, VIC, Australia) to remove any contaminating genomic DNA and stored at −80°C. RNA concentration was determined using a Biospec-nano spectrophotometer (Shimadzu, Sydney, NSW, Australia). RNA was reverse transcribed to cDNA using Omniscript Reverse Transcription kit (Qiagen, Melbourne, VIC, Australia) and stored at −20°C. We performed qRT-PCR using micro fluid cards (Life Technologies) which were pre-customized with Taqman probes (Applied Biosystems, Melbourne, VIC, Australia) for neurogenic differentiation 1 (*Neurod1*-Rn00824571_s1), peroxisome proliferator-activated receptor gamma, coactivator 1 alpha (*Pgc-1*α-Rn00580241_m1), brain derived neurotrophic factor (Bdnf-Rn01484924_m1), nuclear respiratory factor 1 (*Nrf1*-Rn01455958_m1), glycogen synthase kinase 3 alpha (*Gsk3a*-Rn00569232_m1), glycogen synthase kinase 3 beta (*Gsk3b*-Rn00583429_m1), 5-hydroxytryptamine (serotonin) receptor 1A (*Htr1a*-Rn00561409_s1), 5-hydroxytryptamine (serotonin) receptor 2A (*Htr2a*-Rn00568473_m1), v-akt murine thymoma viral oncogene homolog 3 (protein kinase B, gamma; *Akt3*-Rn00442194_m1), v-akt murine thymoma viral oncogene homolog 2 (*Akt*2-Rn00567290_m1), reelin (*Reln*-Rn00589609_m1), nuclear receptor subfamily 3, group C, member 1/glucocorticoid receptor (*Nr3c1*-Rn00561369_m1), homer homolog 1 (*Homer1*-Rn00581785_m1), toll-like receptor 4 (*Tlr4*-Rn00569848_m1), sirtuin 3 (*Sirt3*-Rn01501410_m1), and gamma-aminobutyric acid receptor, subunit alpha 2 (*Gabra2*-Rn01413643_m1). We used 1.5 micrograms of cDNA. 100 microlitres of cDNA/Mastermix (Advanced Fast Mastermix, Life Technologies) mixture was pipetted into the micro fluid cards, after centrifugation the cards were sealed and qRT-PCR was performed. The geometric mean of two housekeepers, tyrosine 3-monooxygenase/tryptophan 5-monooxygenase activation protein (*Ywhaz*) and hypoxanthine phosphoribosyltransferase 1 (*Hprt1*) was used as reference. Analysis was performed using the ΔΔCT method and data expressed relative to an all groups calibrator sample.

### DNA methylation

#### Sodium bisulphite mutagenesis and pyrosequencing

Hippocampal genomic DNA was sodium bisulphite converted with EpiTect kits (Qiagen) according to manufacturer's instructions. Neurod1 promoter was amplified with the primers Neurod1 BisF; ATATAGTTTAGTTGTTGAGGTTGAG, Biotin-Neurod1 Bis R2; BTN-CCACTTTCTTCTAACCACAAAAA. The pyrosequencing primer was Neurod1 pyroseq; TTAGTTGTTGAGGTTGAG and the pyrosequencing sequence to analyse was YGYGGGYGGGGTYGTGAGCT. The GR region primers were GR pyro F; ATTTGGTTTGGGAGGGAAAT, GR pyro R biotin; BTN-CCCCTCTACTAATATAAC and GR pyroseq; AGTTTTTTTTTTTTTAGGT. The sequence to analyse was TTTTTTTYGTTT TYGTTTAGTTTYGAGGTTTGGAATYG. The second CpG that is sequenced in this assay corresponds to the site within the NGFI-A transcription factor binding region at the exon 1_7_ GR promoter that was shown to have methylation altered by maternal behavior (Weaver et al., 2004). For PCR, HotStarTaq (Qiagen) was used and the level of DNA methylation at individual CpG sites in the original sample was ascertained by pyrosequencing on a Pyromark Q96 ID (Qiagen). This technique determines the relative proportions of cytosine (indicating a methylated cytosine) and thymine (indicating an unmethylated, and consequently bisulphite converted cytosine) nucleotides at individual CpG sites in PCR product from regions of interest. Non-CpG C control dispensations were added in each pyrosequencing assay to confirm complete bisulphite conversion of unmethylated cytosine.

### Statistical analysis

Data are presented as mean ± standard error of mean (SEM). Body weight trajectory and average weekly energy intake were analyzed by repeated measures of ANOVA whereas for hippocampal mRNA expression and DNA methylation data were analyzed using Two-way ANOVA (LN exposure and sucrose diet as factors) followed by *post-hoc* Fisher's *LSD*. *p* = 0.05 was considered significant. *Post-hoc* was only conducted when a significant interaction between sucrose consumption and LN exposure was present in the Two-way ANOVA analysis.

## Results

### Effects of sucrose consumption and LN exposure on body weight and energy intake

**Figure 1:**
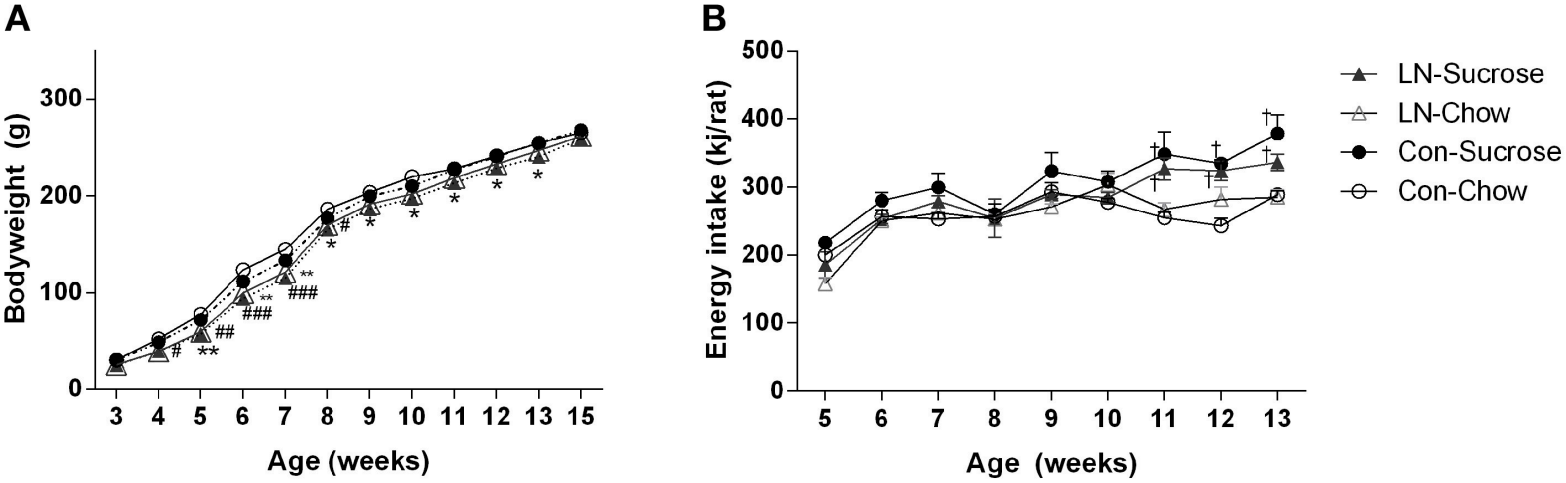
**Body weight trajectory of female pups from weaning to 15 weeks (A) and average weekly energy (kJ/rat) intake from post-weaning week 5–13, ***n*** = 3–4 cages per group (B) for Con-Chow (open circle), LN-chow (open triangle), Con-Sucrose (filled circle), and LN-Sucrose (filled triangle)**. Results are expressed as mean ± S.E.M, *n* = 11–17/group; data were analyzed by repeated measures One-way ANOVA followed by LSD. ^*^p < 0.05, ^**^p < 0.01 vs. control rats (LN effect in chow fed rats). ^#^*p* < 0.05, ^*##*^*p* < 0.01, ^*###*^*p* < 0.001 vs. control rats (LN effect in sugar fed rats). ^†^*p* < 0.05 vs. chow fed rats (diet effect).

In the chow fed group, LN rats were lighter from 4 to 8 weeks of age relative to control rats [Figure 1A; Age × treatment, *F*_(33, 462)_ = 2.303, *p* < 0.0001]. However, in those consuming sucrose, LN rats were lighter from 5 to 13 weeks of age relative to control rats (Figure 1A). Body weight measured when these rats were culled at 15 weeks were similar across groups (*p* > 0.05, see Figure 1A). Relative energy intakes from sucrose were not statistically different across LN and control groups (Con-Suc 1199.8 ± 51.7 vs. LN-Suc 1042.6 ± 76.9 kJ/rat/cage, *p* > 0.05).

Energy intake was measured from 5 to 13 weeks of age (Figure 1B). There were no significant differences in energy intake across groups from 5 to 10 weeks of age. However, rats consuming sucrose from control and LN groups consumed significantly more energy relative to their chow counterparts from 11 to 13 weeks of age [Figure 1B; Age × treatment, *F*_(24, 72)_ = 1.916, *p* = 0.018].

### Effects of sucrose consumption and LN exposure on hippocampal gene expression

**Figure 2:**
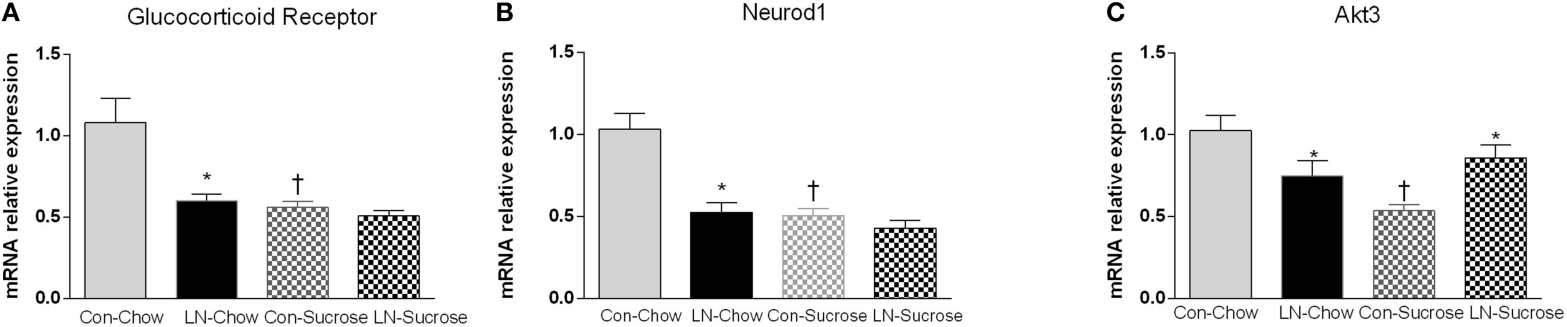
**The effects of LN exposure and sucrose consumption post-weaning on hippocampal gene expression of glucocorticoid receptor (A), Neurod1 (B), and Akt3 (C)**. Results are expressed as mean ± S.E.M; Two-way ANOVA followed by LSD, *n* = 7–8/group. *Post-hoc* analysis was performed when significant interaction between diet and LN was present. ^*^p < 0.05 vs. control rats consuming the same diet (LN effect). ^†^*p* < 0.05 vs. rats consuming chow (diet effect).

The expression of several hippocampal markers related to plasticity and neurogenesis (*Reln, Neurod1, BDNF, Gsk3a, Gsk3b, and GABA-2*), mitochondrial biogenesis (*Akt3/2, Pgc-1*α, *Nrf-1, Sirt-3*), serotonin (*5ht1a, 5ht2a*) and the stress response (*GR, Homer 1*) were analyzed. Diet had a considerable impact on nearly all genes (Table 1). Among these genes, only *Neurod1, GR* and *Akt-3* were significantly altered by LN in the face of the diet consumed. Two-way ANOVA revealed a significant interaction between LN exposure and sugar intake in the expression of *GR* [*F*_(1, 28)_ = 5.660, *p* = 0.024], *Neurod1* [*F*_(1, 26)_ = 11.52, *p* = 0.0022] and *Akt3* [*F*_(1, 28)_ = 14.26, *p* = 0.0008] mRNA in the hippocampus. For *GR*, LN exposure markedly reduced expression by approximately 40% and a similar reduction was observed in control rats consuming sugar (*p* < 0.01, see Figure 2A). There was no further reduction in *GR* mRNA expression by sugar intake in the LN rats (*p* > 0.05, see Figure 2A).

**Table 1:** **Effects of LN exposure and post-weaning sucrose diet on hippocampal expression of genes related to plasticity and neurogenesis, stress response, inflammatory response, mitochondrial biogenesis and serotonin**.

	**Con-Chow**	**LN-Chow**	**Con-Sucrose**	**LN-Sucrose**	**Significance**		
					**Interaction**	**LN**	**Diet**
**NEUROPLASTICITY AND NEUROGENESIS**							
*Reln*	1.04 ± 0.12	1.025 ± 0.10	0.63 ± 0.06	0.46 ± 0.03	ns	ns	*p* < 0.0001
*Gsk3a*	1.03 ± 0.09	0.96 ± 0.06	0.64 ± 0.05	0.56 ± 0.03	ns	ns	*p* < 0.0001
*Gsk3b*	1.03 ± 0.11	0.99 ± 0.08	0.83 ± 0.06	0.77 ± 0.08	ns	ns	*p* = 0.018
*GABA-2*	1.03 ± 0.10	0.84 ± 0.09	0.80 ± 0.09	0.71 ± 0.09	ns	ns	*p* = 0.052
BDNF	1.03 ± 0.07	0.92 ± 0.07	0.97 ± 0.08	0.85 ± 0.07	ns	ns	ns
**STRESS RESPONSE**							
Homer 1	1.01 ± 0.06	0.99 ± 0.06	0.74 ± 0.04	0.70 ± 0.04	ns	ns	*p* < 0.0001
**INFLAMMATORY RESPONSE**							
*TLR4*	1.10 ± 0.19	0.64 ±.0.07	0.89 ± 0.11	0.59 ± 0.06	ns	*p* = 0.002	ns
**MITOCHONDRIAL BIOGENESIS**							
*Pgc-1α*	1.04 ± 0.12	1.18 ± 0.17	0.80 ± 0.07	0.68 ± 0.07	ns	ns	*p* = 0.002
*Akt2*	1.14 ± 0.22	1.36 ± 0.16	0.77 ±.0.08	0.84 ± 0.09	ns	ns	*p* = 0.004
*Nrf-1*	1.01 ± 0.06	0.95 ± 0.10	0.75 ± 0.05	0.57 ± 0.05	ns	ns	*p* < 0.0001
*Sirt-3*	1.01 ± 0.07	1.15 ± 0.14	0.92 ± 0.07	0.81 ± 0.07	ns	ns	*p* = 0.026
**SEROTONIN**							
5ht1a	1.02 ± 0.09	1.17 ± 0.05	0.72 ± 0.05	0.75 ± 0.08	ns	ns	*p* < 0.0001
5ht2a	1.04 ± 0.12	1.27 ± 0.11	0.85 ± 0.09	0.94 ± 0.16	ns	ns	ns

Results are expressed as mean ± S.E.M; Two-way ANOVA followed by LSD, n = 7–8/group. Post-hoc analysis was performed when significant interaction between diet and LN was present. ^*^p < 0.05 vs. control rats consuming the same diet (LN effect). ^†^p < 0.05 vs. rats consuming chow (diet effect).

For *Neurod1*, LN exposure significantly reduced mRNA expression by about 46% (*p* < 0.01, see Figure 2B) and sugar intake similarly affected the expression of hippocampal *Neurod1* in the control rats (*p* < 0.01, see Figure 2B). However, no further reduction was observed in LN rats consuming sugar (*p* > 0.05, see Figure 2B).

For *Akt3*, both sucrose consumption and LN exposure reduced mRNA expression; however sucrose consumption appeared to exhibit a greater reduction (50 vs. 25%, respectively). Interestingly, in those consuming sucrose, control rats had significantly lower expression of *Akt-3* mRNA relative to LN rats (*p* < 0.05, see Figure 2C).

### Effects of sucrose consumption and LN exposure on DNA methylation of GR AND Neurod1 promoter regions

**Figure 3:**
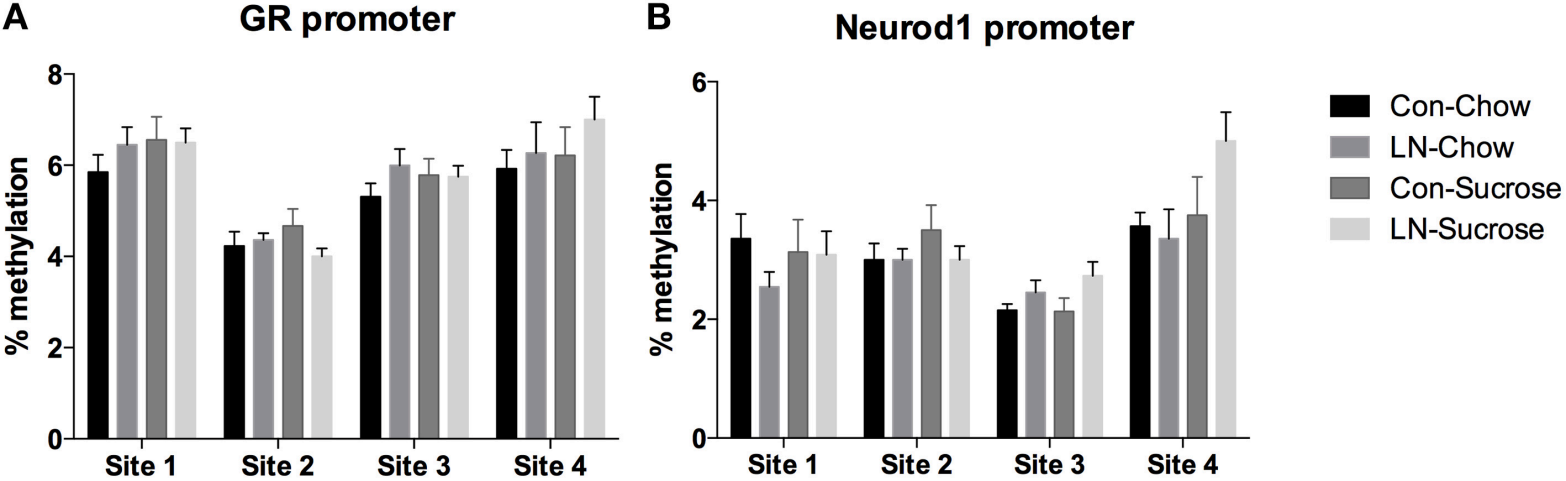
**Percentage of DNA methylation at 4 CpG sites in the (A) ***GR*** and (B) ***Neurod1*** promoter regions across Con, LN, Con-Sucrose, and LN-Sucrose groups**. Results are expressed as mean ± S.E.M; Two-way ANOVA followed by LSD, *n* = 8–13/group. No significant interaction was observed between LN exposure and sucrose diet.

The contribution of DNA methylation to the effects of LN and post-weaning sugar intake on *GR* and *Neurod1* mRNA expression was assessed by examining DNA methylation at 4 CpG sites in the *GR* (Figure 3A) and *Neurod1* (Figure 3B) promoters. Two-way ANOVA analysis revealed that there were no significant interactions between LN exposure and post-weaning sucrose intake across all of the 4 CpG sites in the *GR* (*p* > 0.05, see Figure 3A) and *Neurod1* (*p* > 0.05, Figure 3B).

## Discussion

This study demonstrated that in female rats, early life stress in the form of LN had a long-lasting effect on hippocampal gene expression particularly *GR* and *Neurod1* which have important roles in stress regulation and neurogenesis, respectively. The novelty of this study lies in the finding that chronic consumption of sugar produced equivalent hippocampal molecular deficits as early life stress exposure in the form of LN. Contrary to our hypothesis, we found no evidence for the marked reduction of *GR* and *Neurod1* mRNA by LN exposure and sucrose intake being mediated by promoter DNA methylation. This study provides the first evidence of LN exposure in combination of post-weaning sucrose consumption on hippocampal gene expression in females and whether similar effects occur in males is yet to be explored.

There is a strong body of evidence showing that early life stress leads to persistent alterations in expression of hippocampal genes related to stress regulation and plasticity during adulthood (Murgatroyd et al., 2009). For example, *GR*, a key regulator of the HPA axis activity was reported to be altered by early life stress in both human (McGowan et al., 2009) and animal (Maniam and Morris, 2010a,b) studies. Previous work from our lab showed that early life stress using maternal separation reduced hippocampal *GR* mRNA by about 20% in both male and female rats (Maniam and Morris, 2010a,b), however, here LN exposure reduced its expression by 40% in female rats. Interestingly, while high sucrose diet also led to a marked reduction of *GR* mRNA in the control group, the diet led to no further reduction in the LN rats. This suggests that the high sugar diet produced an equivalent deficit in expression of *GR* mRNA in the hippocampus. While there are several lines of evidence demonstrating that drinks enriched with sugar inhibit stress induced avoidance behavior (Minor and Saade, 1997; Dess et al., 1998), the consequences of such a diet on hippocampal *GR* expression is not known. There is some evidence for effects of combined sugar and fat enriched diets on hippocampal *GR* expression. For example, increased expression of *GR* mRNA in the CA1 region of the hippocampus was observed in dietary obesity and these rats were less anxious compared to chow fed rats, with increased activity in the open field test (Michel et al., 2003). Taken together, there appears to be differential effects of sugar and fat diets on hippocampal *GR* expression. As reduction in hippocampal *GR* mRNA expression was reported in depression and suicide subjects (López et al., 1998), *GR* can be a molecular marker for mental health disorders. Given that sucrose consumption led to similar reduction in hippocampal *GR* mRNA expression in this study, we postulate that sucrose consumption may increase the risk for psychiatric disorders to a similar degree to those exposed to early life stress. Moreover, as these two factors often co-exist in the general population, interventions to offset these deficits observed at the molecular level are critical.

Another gene that was impacted by both sucrose and LN exposure was *Neurod1*. *Neurod1* is a downstream regulator of neurogenesis that modulates the expression of genes critical for maturation and neuronal functionality such as structural proteins and ion channels, and coordinates terminal differentiation in dentate gyrus granule cells (Schwab et al., 2000). Given neuronal migration is critical for CNS development (Gao et al., 2009), *Neurod1* is required for the survival of the hippocampal dentate gyrus as evidenced by reduced dentate gyrus cell numbers with deletion of *Neurod1* (Kim, 2013) which is in line with an earlier study (Miyata et al., 1999). The expression of *Neurod1* is reported to occur at a later stage of CNS development (Cho and Tsai, 2004). While *Neurod1* is a critical gene for adult neurogenesis, the contribution of this gene to early life stress induced neurogenesis deficits is not known. Here, we provide the first evidence that early life stress in the form of LN halved the hippocampal expression of this gene. Early life stress exposure is widely known to reduce adult neurogenesis (Karten et al., 2005; Korosi et al., 2012; Lajud and Torner, 2015; Naninck et al., 2015). The strong link between *Neurod1* and neurogenesis combined with the marked deficit in the expression of hippocampal *Neurod1* in this study further suggest a potential role of *Neurod1* in mediating early life stress induced alterations in neurogenesis and associated cognitive deficits such as those recently reported (Suri et al., 2013; Naninck et al., 2015). Strikingly, in our hands, sucrose consumption in control female rats had a similar effect on *Neurod1* mRNA expression. This finding further suggests that chronic sucrose consumption may affect adult hippocampal neurogenesis and increase risk for hippocampal dependent memory deficits. While we and others have consistently shown in previous work that acute and chronic consumption of liquid sugar impairs spatial recognition memory (Beilharz et al., 2014, 2015), the role of *Neurod1* in mediating these effects is yet to be explored. Hence, this should be the focus of future studies.

*Akt3* was also affected by LN exposure and chronic sucrose consumption in female rats. Interestingly, sucrose alone produced marked reductions in *Akt3* mRNA and this deficit in expression was greater when compared to LN rats consuming the same diet. *Akt3*, also known as protein kinase B (PKB), is a critical signaling molecule in the phosphatidylinositol 3 kinase (PI3k) pathway involved in an array of cellular processes including survival, growth, proliferation, metabolism and migration (Easton et al., 2005). Increased Akt expression has been associated with increased neurogenesis and neuroprotection after brain injury and seizures (Wu et al., 2008a,b; Piermartiri et al., 2009). *Akt3* is the predominant Akt isoform in the adult mammalian brain which accounts about 50% of the *Akt* in the hippocampus (Easton et al., 2005). *Akt3* in particular has been associated with postnatal brain development as evidenced by a reduction of brain size by about 25% in *Akt3* null mice (Easton et al., 2005; Tschopp et al., 2005). Hence, the reduced expression of *Akt3* mRNA by sucrose and LN exposure suggests increased risk of adverse brain development which needs to be systematically examined in future work in both male and females. There is some evidence of novel stress (restraint stress) exposure in rodents affecting brain PI3K- Akt related transcripts (Barreto et al., 2012). However, to the best of our knowledge, there appears to be no other reports of early life stress impacting *Akt* gene/protein expression or activation. The effect of high energy diets, such as sucrose and high fat diet on the Akt pathway in the periphery is well defined, however, the consequences of such diets on the central Akt pathway is unclear. Increases in insulin and glucose, which commonly occur with chronic consumption of diets high in sugar or fat (Maniam and Morris, 2010a,b), enhance phosphorylation of *Akt* in the brain (Clodfelder-Miller et al., 2005). As we only measured mRNA expression, it is unknown whether activity of Akt was affected by LN exposure or the combination of diet. This is an important question to explore given Akt activity is upstream of several targets involved in cell growth, proliferation and apoptosis.

While we observed a marked effect on hippocampal gene expression relevant to synaptic plasticity and stress response (*GR, Neurod1*, and *Akt3*), there are several other synaptic and neurogenesis genes (*GSKBa, GSKBb, Reln*), mitochondrial biogenesis genes (*Pgc-1*α*, Akt2, Nrf1, Sirt-3*) and serotonin related gene (*5Ht1a*) which were affected by sucrose consumption as evidenced by a main effect of sucrose consumption in the Two-way ANOVA analysis. This is the first study to demonstrate the impact of chronic sucrose consumption on an array of genes that govern development, emotional and functionality of the brain. There is increasing evidence regarding the impact of combined high fat and sugar diet on hippocampal plasticity and inflammatory genes (Molteni et al., 2002; Stranahan et al., 2008; Beilharz et al., 2015) with limited evidence on the impact of liquid sugar on hippocampal genes (Beilharz et al., 2015). To the best of our knowledge, our results are the first evidence for a high sucrose diet affecting hippocampal expression of genes associated with mitochondrial biogenesis as evidenced by a main effect of diet on *Pgc-1*α mRNA expression. One recent study did report an effect of chronic high fat diet feeding on hippocampal Pgc-1α protein in mice. Six months of high fat almost halved the expression of hippocampal Pgc-1α protein compared to chow (Petrov et al., 2015). Importantly, this data are also one of the few studies reporting effects in females.

There is emerging evidence showing that early life stress-induced hippocampal gene expression changes are mediated by epigenetic changes (McGowan et al., 2009; Roth et al., 2009). Several epigenetic processes may underlie the changes in gene expression induced by early life stress; however, one particular mechanism that has been recently well-studied is DNA methylation. The first epigenetic evidence underlying early adversity (low maternal care) induced reduction of *GR* mRNA was mediated by hypermethylation of hippocampal *GR* mRNA (GR exon 1_7_ promoter; Weaver et al., 2004). However, here we show for the first time that a marked reduction of hippocampal *GR* mRNA was not associated with altered DNA methylation across the same region of the *GR* promoter. Several differences in the experimental set-up exist between our LN and that of Weaver et al. (2004), including, the quality of maternal care imposed by LN vs. natural maternal nursing behavior and the rat strain used (Sprague-Dawley vs. Long-Evans hooded). Both models are linked to altered maternal behavior with reduced GR expression in adult offspring. LN exposure has been reported to affect quality of maternal care provided to the offspring; LN dams exhibit fragmented care to their pups (Ivy et al., 2008). Our data therefore show that altered DNA methylation at the *GR* exon 1_7_ promoter is not a prerequisite for early-life environment-programmed reduction in *GR*.

The *Neurod1* mRNA expression pattern was similar to that of *GR*, in that LN and sucrose both reduced it. *Neurod1* induces terminal neuronal differentiation during neurogenesis (Boutin et al., 2010). *Neurod1* has been shown to have increased methylation levels in a variety of cancers (Fiegl et al., 2008; Selamat et al., 2011), while variation in the gene locus methylation state has been observed in human brain (Siegmund et al., 2007). However, no previous studies have investigated whether dietary- or stress-induced transcriptional changes of *Neurod1* are associated with DNA methylation changes at the promoter. In our novel pyrosequencing assay we found no evidence for DNA methylation changes due to LN or sucrose diet at the *Neurod1* promoter. The lack of DNA methylation changes at GR and *Neurod1*, which both have reduced expression due to LN and sucrose, may suggest that other epigenetic mechanisms such as histone-tail modifications cause transcriptional change. Indeed it is likely that promoter histone acetylation is a key regulator of *Neurod1* transcriptional regulation in neurogenesis. An epigenetic switch between neural stem cells and neural progenitor cells has been shown to involve repression of *Neurod1* in the former with the Sox2/HDAC1 (histone deacetylase 1) repressor complex and activation in the latter by a β-catenin/TCF/LEF activator complex, following Wnt activation (Gao et al., 2009). Of course we cannot exclude the possibility that there may be DNA methylation changes at other regions of the *GR* and *Neurod1* genes. The absence of a positive control for detecting methylation change at *GR* and *Neurod1* means that we are unable to exclude the possibility that the absence of methylation changes are due to a technical reason. However, we have previously successfully used sodium bisulphite pyrosequencing to detect diet-induced DNA methylation changes (Youngson et al., 2015), and the promoter regions that we chose to investigate have both been shown to have variable methylation (Weaver et al., 2004; Siegmund et al., 2007; Fiegl et al., 2008; Selamat et al., 2011).

In summary, LN exposure or post-weaning sucrose consumption reduced hippocampal *GR* and *Neurod1* mRNA expression to a similar degree in female rats. If similar effects occur in humans, early life adversity and high sugar diet may independently increase the risk for psychopathology later in life. While DNA methylation has been associated with early life stress induced hippocampal DNA methylation, here we found no evidence for such contribution to the changes of hippocampal *GR* and *Neurod1* mRNA expression following LN exposure and chronic sucrose consumption post-weaning. The similarity in the hippocampal molecular deficits induced by sugar and early life stress is of great concern given the cheap and easy accessibility of sugar-sweetened beverages. While the combined poor diet and early life stress did not produce further hippocampal molecular deficits, whether this remains the case as the rats age is unclear and is an important future question. If similar processes are at play in humans, manipulating the later environment of those exposed to early life adversity, and controlling the consumption of sugar-sweetened beverages across the community may be an effective way to curtail the burden of psychiatric disorders.

## Author contributions

Designed the experiments: JM and MM. Performed the experiments: JM, CA, NY, and JS. Analyzed the data: JM, CA, NY, and JS. All authors contributed to the writing of the manuscript.

### Conflict of interest statement

The authors declare that the research was conducted in the absence of any commercial or financial relationships that could be construed as a potential conflict of interest.
